# Brain Networks Involved in Depression in Patients with Frontotemporal Dementia and Parkinson’s Disease: An Exploratory Resting-State Functional Connectivity MRI Study

**DOI:** 10.3390/diagnostics12040959

**Published:** 2022-04-12

**Authors:** Vincenzo Alfano, Giovanni Federico, Giulia Mele, Federica Garramone, Marcello Esposito, Marco Aiello, Marco Salvatore, Carlo Cavaliere

**Affiliations:** 1 Istituto di Ricovero e Cura a Carattere Scientifico (IRCCS) Synlab SDN, Via Emanuele Gianturco, 113, 80143 Naples, Italy; vincenzo.alfano91@gmail.com (V.A.); giulia.mele@synlab.it (G.M.); garramonefederica@gmail.com (F.G.); marco.aiello@synlab.it (M.A.); direzionescientifica.irccssdn@synlab.it (M.S.); carlo.cavaliere@synlab.it (C.C.); 2 Azienda Ospedaliera di Rilievo Nazionale (AORN) Antonio Cardarelli, 80131 Naples, Italy; marcelloesposito@live.it

**Keywords:** depression, fMRI, frontotemporal dementia, Parkinson’s disease, neuropsychology

## Abstract

Depression is characterized by feelings of sadness, loss, or anger that may interfere with everyday activities. Such a neuropsychiatric condition is commonly reported in multiple neurodegenerative disorders, which are quite different from each other. This study aimed at investigating the brain networks involved in depression in patients with frontotemporal dementia (FTD) and Parkinson’s disease (PD) as compared to healthy controls (HC). Fifty participants were included in the study: 17 depressed FTD/PD patients; 17 non-depressed FTD/PD patients; and 16 non-depressed HCs matched for age and gender. We used the Beck depression inventory (BDI-II) to measure depression in all groups. On the same day, 3T brain magnetic resonance with structural and resting-state functional sequences were acquired. Differences in resting-state functional connectivity (FC) between depressed and non-depressed patients in all the experimental groups were assessed by using seed-to-seed and network-to-network approaches. We found a significant seed-to-seed hyperconnectivity patterns between the left thalamus and the left posterior temporal fusiform cortex, which differentiated FTD/PD depressed patients from the HCs. Network-to-network analysis revealed a significant hyperconnectivity among the default-mode network (left lateral-parietal region), the medial prefrontal cortex and the left lateral prefrontal cortex (i.e., part of the central executive network). We investigated whether such FC patterns could be related to the underlying neurodegenerative disorder by replicating the analyses with two independent samples (i.e., non-depressed PD and non-depressed FTD patients) and adding clinical parameters as covariates. We found no FC differences in these groups, thus suggesting how the FC pattern we found may signal a common depression-related neural pathway implicated in both the neurocognitive disorders.

## 1. Introduction

Depression is a neuropsychiatric condition characterized by feelings of sadness, loss, or anger that may interfere with individuals’ everyday activities. Depression is one of the leading causes of disability worldwide, with a lifetime prevalence of 11% in European countries [1]. Depression is commonly reported in many neurodegenerative disorders, such as Alzheimer’s disease (AD), mild cognitive impairment (MCI), and Parkinson’s disease (PD) [2,3,4]. Most interestingly, mood disorders have also been reported in a completely different class of disorder, namely in patients with frontotemporal dementia (FTD) [5,6,7]. Thus, while being quite different from each other, these neurodegenerative disorders seem to recognize depression as a possible shared comorbidity. The absence of treatments and their high prevalence has led, over the last years, to intense research efforts to investigate the neurobiological bases of depression [8]. In the last decades, neuroimaging research obtained a relevant role in highlighting how depression may be described as a multifactorial disorder, which includes abnormal brain activity and structural alterations of several brain regions [9,10]. Indeed, by using functional magnetic resonance imaging (fMRI), researchers can measure the brain activity during the “resting state”, namely when participants perform no tasks [11]. The main large-scale brain networks investigated in depression are the default-mode network (DMN), the salience network (SN), and the frontoparietal network or central executive network (CEN) [10]. The main regions of interest (ROIs) in DMN are the medial prefrontal cortex (MPFC), the posterior cingulate cortex or precuneus, and both the left and right inferior parietal cortex (LPC). DMN is active when a person is not focused on the outside world and the brain is at wakeful rest. Also, DMN is important for affective and cognitive processes [9]. The main ROIs in SN are both the left and right insular cortices as well as the anterior cingulate cortex. Such a network is crucial for the detecting and filtering of salient stimuli salient information and for switching between the DMN and CEN [12,13]. The main ROIs in CEN are both the left and right lateral prefrontal (LPFC) and both the left and right posterior parietal cortices. CEN supports executive and higher-level cognitive functioning [14]. Recent research highlighted that DMN, SN, and CEN’s FC may be impaired in depression. For instance, both intra-networks and between-networks alterations are reported in the literature, with various effects and different hypo/hyperconnectivity patterns in distinct brain regions [15,16]; most interestingly, increased DMN FC has been reported in depressed patients. This observation has been linked to the recursive effects of their symptoms and their excessive tendency to self-focusing [17,18].

Another region often involved is represented by the thalamus and its networks. The thalamus plays an important role in cognitive and emotional processes due to its interactions within prefrontal–temporal, prefrontal–amygdala, and prefrontal–basal ganglia networks [19]. Moreover, the thalamus modulates sensory information from peripheral sensory organs to sensory cortices (first-order relay) as well as information between cortical regions (higher-order relay) [19,20]. Activity within these networks demonstrates both top-down and bottom-up modulation of emotion, motivation, drive, and attention [21,22], which can all be impacted by depression [23,24]. Finally, to support these modulations, a clinical study has documented the association between specific thalamic damage and cognitive/emotional deficits [25].

FTD and PD patients may experience visual–cognition deficits associated with dementia, which may reflect the effects of distinct fronto-parieto-temporal impairments [26,27,28,29]. Indeed, most recent studies highlighted how high-level visual cognition may rely on the interplay of wide fronto-temporo-parietal brain networks, which integrate distinct kind of information (i.e., semantic, mechanical, and sensorimotor knowledge) in the context of daily activities [30,31]. These networks can be selectively or globally disrupted in neurodegenerative disorders [32,33,34]. Accordingly, PD and FTD patients may encounter difficulties in visual-spatial abilities and high-level visual-cognition tasks such as facial processing and object recognition [26,28]. Also, high-level visual cognition might be critically involved in emotional processing and social interactions. In the clinical context, therefore, high-level visual-cognition deficits may concur in exacerbating depressive symptoms [35]. Recent advances in cognitive neuroscience have indicated that in the context of a complex occipito-temporo-parietal network, the fusiform gyrus (FG) brain region is critically involved in high-level visual cognition, particularly in face processing [36,37]. Congruently, fMRI studies highlighted how impairments in FG may produce visual–cognition alterations during face-matching tasks [38,39]. In addition, AD and MCI patients, as compared to healthy controls, may exhibit functional activation changes in FG during visual working memory tasks [40]. However, most recent research links visual–spatial dysfunctions more clearly to dementia than depression and parietal dysfunctions, and much less with the fusiform gyrus [28,29]. Thus, visual–cognition symptoms might not be related directly to FG dysfunctions. Indeed, they might emerge as an effect of impairments in distinct linked-with-FG brain regions [37].

Most neuroimaging studies involving depressed FTD and PD patients considered structural and metabolic changes, thus identifying changes in cortical thickness, white matter integrity, perfusion, and metabolism [7,41,42]. However, FC data about common pathways of depression in both FTD and PD is still lacking. For this reason, this study’s primary aim is to characterize the neural underpinnings of depression in these neurodegenerative disorders, which are completely different from each other. Therefore, in this study, we considered PD and FTD as opposite classes of disease that share depression as the most common neuropsychological condition. Thus, in the present exploratory study we aimed to investigate large-scale brain networks involved in these conditions by using integrative and quantitative functional connectivity analyses.

## 2. Materials and Methods

Fifty participants were enrolled in the study. Specifically, 17 patients with depression syndrome: 9 with a primary diagnosis of FTD (mean age 63.2 ± 7.3) and 8 with a primary diagnosis of PD (mean age 65.5 ± 9.2); 17 non-depressed patients: 8 FTD (mean age 70.1 ± 5.6) and 9 PD (mean age 64.2 ± 7.1); 16 healthy and non-depressed controls (HC) matched for age and sex. The study was conducted at the IRCCS Synlab SDN and included a clinical evaluation of depression and a 3T magnetic resonance imaging (MRI) protocol. All participants were recruited if they met the following criteria: (i) lack of current or past history of alcohol or drug abuse, (ii) lack of current or past use of psychoactive medications. All participants were assessed by both an expert neurologist and neuropsychologist according to DSM-V [43]. Patients with incidental brain focal lesion to MRI examination or excessive vascular load were excluded [44]. Beck depression inventory (BDI-II) [45] was used to investigate and measure the presence of depression in FTD, PD and HC groups (range 0–63, cutoff value for mild depression: 20). Mini-mental state examination (MMSE) [46] was used to measure cognitive impairment in FTD, PD and HC groups. Unified Parkinson’s disease rating scale (UPDRS) [47] was used to follow the longitudinal course and the severity of PD in the PD group. Both demographic and neuropsychological information are resumed in Table 1. Each participant provided written informed consent. The local Ethics Committee (IRCCS Pascale) approved the study following ethical standards laid down in the 1964 Helsinki Declaration.

MRI was acquired by using a Biograph mMR 3T scanner (Siemens Healthcare, Erlangen, Germany). A 12-channel head coil was used in a customized neurological protocol including the following structural and functional sequences: (1) 3D T1-Magnetization Prepared Rapid Acquisition Gradient Echo (MPRAGE), voxel size 0.8 × 0.8 × 0.8 mm^3^, field of view (FOV) 214 × 214 mm, TR/TE/TI = 2400/2.25/1000 ms, scan time 5:03; (2) resting-state fMRI, sequence Echo Planar Imaging-Gradient Echo (EPI-GRE), voxel-size 4 × 4 × 4 mm^3^, TR/TE = 1000/21.4 ms, 350 measurements, bandwidth: 2230 Hz, scan time 6:02.

For structural image processing, the parcellations of morphological T1-weighted 3D images of HC and the PD, FTD groups were processed with the FreeSurfer v5.1 toolkit [48]. This processing includes spatial inhomogeneity correction, non-linear noise reduction, skull-stripping, subcortical segmentation, intensity normalization, surface generation, topology correction, surface inflation, registration to a spherical atlas, and cortical thickness calculation [49]. Consequently, the result was normalized by the ratio with the estimated total intracranial volume (eTIV). Then, a two-tailed two-sample *t*-test, corrected for Bonferroni multiple comparisons (significant *p*-value < 0.0004), was performed with SPSS 26 (IBM Statistics) to compare brain morphological parameters (cortical volume and cortical thickness) between groups.

fMRI data were analyzed by using the functional connectivity toolbox (CONN) 21b [50] and SPM 12 software (Statistical Parametric Mapping: The Analysis of Functional Brain Images). Preprocessing was carried out in CONN using a pipeline that includes realignment, slice-timing, functional image normalization by using the Montreal Neurological Institute (MNI) reference space, outlier detection with ART-based scrubbing, 8 mm smoothing, and physiological denoising. Finally, additional steps included detrending, despiking, and filtering (0.008 Hz < f < 0.09 Hz) to the residual time series.

A first-level statistical analysis was conducted to assess subjects’ resting-state brain activations. Then, a second-level data analysis was devised to assess FC differences among the three groups. First, we evaluated FC differences among depressed patients, non-depressed patients and HCs by performing a CONN-based seed-to-seed analysis. Then, two seed-to-seed analyses (i.e., FTD vs. HC, and PD vs. HC) were performed to investigate whether FC differences were due to depression as a trait or belong to a primary pathological condition. Finally, to validate the FC differences in depressed patients, the same analysis was carried out between non-depressed patients (FTD-PD groups) and HCs. A *p*-value of 0.05 corrected for false discovery rate (FDR) multiple comparisons [51] was considered significant for FC analysis. We included in the second-level analysis age, gender, MMSE, and UPDRS as covariates in order to test if these factors were related to the FC results. In addition, we included a correlation analysis to investigate the relationship between depression symptoms (BDI score) and clinical parameters (MMSE and UPDRS). CONN seed-to-seed analyses were conducted by adopting (1) a cortical and subcortical ROI-to-ROI approach (FSL Harvard–Oxford maximum likelihood cortical and subcortical atlas, dividing bilateral areas into left/right hemisphere for a total of 106 ROIs); (2) a network-to-network approach (from CONN’s ICA analyses of HCP dataset for a total of 8 networks with 32 subnetwork ROIs).

## 3. Results

**Figure 1:**
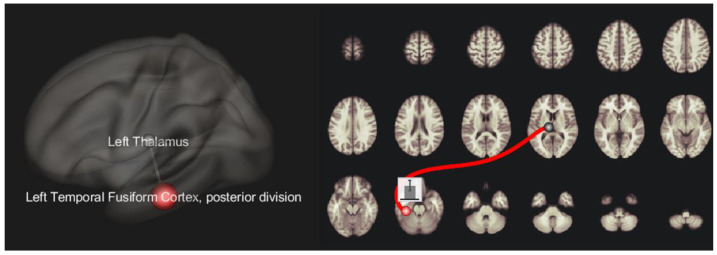
fMRI seed-to-seed 3D representation showing the hyperconnectivity between left thalamus and left posterior temporal fusiform cortex.

The structural analysis did not show significant differences in brain parcels volumes and cortical thickness between depressed patients, non-depressed patients and HCs. The resting-state paradigm showed a significant difference between depressed patients (both FTD and PD) and HCs; the same pattern was also confirmed between depressed and non-depressed patients (both FTD and PD), hence highlighting a seed-to-target hyperconnectivity between left Thalamus and left posterior temporal fusiform cortex (p-FDR = 0.01). This result was confirmed when the primary diagnosis was revealed in the depressed group in seed-to-seed analysis between depressed FTD patients and HC (p-FDR = 0.05), and between depressed PD patients and HC (p-FDR = 0.04). T-score and *p*-value FDR corrected in these seeds are resumed in Table 2 and Figure 1.

**Figure 2:**
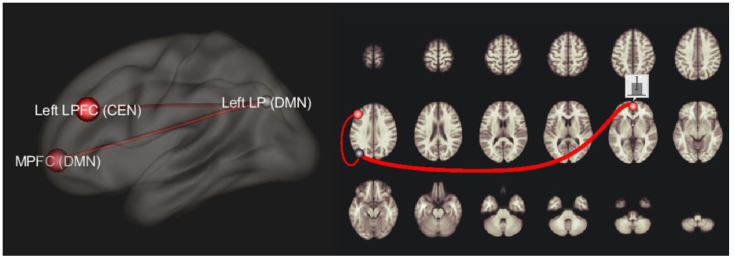
fMRI network-to-network 3D representation showing the hyperconnectivity between the left lateral parietal cortex (LP), left lateral prefrontal cortex (LPFC), and medial prefrontal cortex (MPFC).

The network-to-network analysis highlighted a significant difference between depressed patients and HCs, namely a hyperconnectivity between left DMN (lateral parietal part) as seed and DMN MPFC (p-FDR = 0.03) and left LPFC (p-FDR = 0.03) as targets. This result was also confirmed when the primary diagnosis was revealed in the depressed group in network-to-network analysis between depressed FTD patients and HC (p-FDR = 0.01 for MPFC and p-FDR = 0.05 for LPFC) and between depressed PD patients and HC (p-FDR = 0.05 for MPFC and p-FDR = 0.05 for LPFC). T-score and *p*-value FDR corrected in these seeds are resumed in Table 3 and Figure 2. By including age and sex as covariates did not change these FC results, while MMSE on FTD and PD, and UPDRS only for PD affected the results. These patterns across depressed patients were not confirmed when considering non-depressed FTD-PD patients vs. HC. A negative correlation (r = −0.37, *p* < 0.01) between BDI and MMSE scores was found in both FTD and PD groups. FC correlates only with MMSE in FTD groups when left LP and left LPFC were considered as seeds (r = 0.42, *p* < 0.01). No correlations were found in other FC ROIs and networks with MMSE.

## 4. Discussion

As the main finding of this study, we found that depressed patients have a significant increase in functional brain connectivity in the following regions: left thalamus with left fusiform cortex, and within DMN for the connectivity of left lateral parietal part with MPFC or left LPFC. Our results highlight a common pathway for depression in both FTD and PD patients, thus suggesting the involvement of specific large-scale brain networks as a shared neural substrate for these disorders.

Current research addressing the neuropathological mechanisms of depression is mainly focused on the widely recognized limbic-cortical-striatal-thalamic circuit [52]. This evidence suggests that depression is closely linked with the morphology and function of this circuit [53], for instance, the prefrontal cortex, anterior cingulate cortex, basal ganglia, thalamus, hippocampus, and amygdala volume is reduced in depressed patients [54,55,56]. In our study, we did not find significative structural alterations between depressed patients, non-depressed patients, and HCs, probably due to sample differences in disease severity, medication, gender, and familiarity of mental illness that could bias the analyses [57]. Some studies of functional connectivity have also revealed abnormal connections in areas related to the limbic–cortical–striatal–thalamic circuit. For instance, FC between the subgenual cingulate and the thalamus are enhanced in depression [58]. According to a previous study, the thalamus is a pivotal site that integrates various neural activities from widespread cortical inputs and outputs and is considered to modulate communication with brain regions [59]. Indeed, the left thalamus is hyperconnected with left FG in both FTD and PD patients, hence highlighting how its role may be crucial in the depression associated with these neurodegenerative diseases. Previous neuroimaging studies found significant connectivity between the thalamus and several brain regions, such as the frontal, temporal, parietal, and occipital lobes [60,61,62]. Some of these connections, such as the thalamus–fusiform connection [63], are considered to be important pathways for visual memory processing, considering the thalamus involvement in selective attention and visual discrimination [64]. In a study with MCI patients, the increased connectivity between these two regions has been interpreted as the recruitment of additional neural resources to compensate for losses of cognitive functions [37]. Such a neurocognitive framework is consistent with our results, which show greater FC between the thalamus and the posterior FG, in patients with a neurodegenerative disease such as FTD and PD and in patients with depression. Moreover, our result is also consistent with the hypothesis of disturbed thalamocortical connectivity across highly specific and localized regions in patients with depression [20]. Finally, greater thalamo–temporal connectivity was associated with more severe depressive symptoms, suggesting an association with the core psychopathology of depression [20]. Our result on FTD and PD patients is in line with other neuroimaging studies that show a positive relationship between thalamic activity and both depression symptoms [65] and treatment-resistant depression [66].

As the second major highlight provided by our results, we found a FC hyperconnectivity pattern among the left DMN (lateral parietal region) and both MPFC and left LPFC (CEN region). Intriguingly, the left LPFC is responsible for top-down voluntary modulation of both positive and negative emotions [67]. Furthermore, a study applying fast TMS over the left LPFC demonstrated an antidepressant effect [68]. Sheline and colleagues [69] found a frontal area, namely the “dorsal nexus” area (DN), to show aberrant FC with nodes of the DMN, CEN, and SN in depression and hypothesized that this area “hot-wires” the three networks together, leading to various depressive symptoms. DN overlaps with the left LPFC and MPFC ROIs used in our study. In line with the gateway hypothesis and findings regarding the DN, we found a specific alteration between the DMN, a network that is associated with internal mental processes, and the CEN, a network crucial for processing external inputs [9,12,13,14]. Notably, a recent study underlined how reduced FC within the DMN in depressed individuals, as compared to HCs, may reflect the use of medications rather than illness duration [70]. This is in line with our findings that report greater FC within the DMN in unmedicated depressed patients. Finally, altered FC between the DMN and CEN may underlie an impairment in switching from a “default state”, with attention directed to internal mental processes, to an “executive state”, in which attention is assigned to external stimuli [71,72,73]. Moreover, correlation analyses between clinical scores and FC findings, using MMSE and UPDRS as covariates, demonstrated a relationship between depression and cognitive impairment and/or disease severity. This result could be related to differences in our subgroups and should be confirmed with further studies.

To conclude, in this study, we identified depression-related brain networks, which are potentially shared by completely different classes of disorders (i.e., FTD and PD). While being an exploratory study with some limitations, such as the non-inclusion of a fourth group of depressed HCs and a limited sample size, the findings we reported may constitute an important step toward our understanding of alterations in large-scale brain networks involved in depression across distinct neurocognitive disorders. Furthermore, our results underline the role of thalamocortical and DMN-CEN systems in cognitive and emotional information processing and how they may be associated with depression in heterogeneous clinical samples. However, while our indications may stimulate clinical reflections, further studies with larger samples should elaborate on our preliminary findings.

## Figures and Tables

**Table 1:** Demographic and neuropsychological data in the five groups. (depFTD: depressed FTD patients; depPD: depressed PD patients; non-depFTD: non-depressed FTD patients; non-depPD: non-depressed PD patients; HC: healthy controls. Age and education expressed in years).

	depFTD		depPD		Non-depFTD		Non-depPD		HC	
	Mean (SD)	Range	Mean (SD)	Range	Mean (SD)	Range	Mean (SD)	Range	Mean (SD)	Range
**Age**	63.2 (7.3)	53–75	65.5 (9.2)	63–80	70.1 (5.6)	64–81	64.2 (7.1)	52–71	57.7 (6.7)	48–70
**Gender**	4F–5M	\	4F–4M	\	2F–6M	\	4F–5M	\	7F–9M	\
**Education**	9.2 (3.4)	5–13	10.1 (3.6)	8–18	15.4 (3.7)	8–18	11.2 (3.7)	8–18	14.9 (3.5)	8–18
**BDI**	31.1 (10.2)	20–54	24.2 (5.6)	20–37	9.6 (4.8)	2–17	3.7 (2.3)	1–6	6.6 (4.6)	1–16
**MMSE**	22.9 (5.8)	14–29	23.6 (6.1)	13–29	27.6 (2.3)	24–30	27.4 (1.5)	25–30	28.5 (1.4)	27–30
**UPDRS**	\	\	33.2 (10.9)	17–50	\	\	20.1 (8.6)	11–36	\	\

**Table 2:** Resting-state fMRI seed-to-seed findings between depressed patients and HC group and between depressed FTD and PD patients and HC (higher connectivity between seeds have a positive value of T-score; p-FDR: *p*-value corrected for false discovery rate).

Seed	Target	T-Score	p-FDR
	**Depressed patients > HC**		
Left thalamus	Left posterior-temporal fusiform cortex	4.2	0.01
	**Depressed FTD > HC**		
Left thalamus	Left posterior-temporal fusiform cortex	3.6	0.05
	**Depressed PD > HC**		
Left thalamus	Left posterior-temporal fusiform cortex	3.6	0.04

**Table 3:** Resting-state fMRI network-to-network findings between depressed patients and HC group and between depressed FTD and PD patients and HC (higher connectivity between seeds have a positive value of T-score; LP: lateral parietal lobule; DMN: default-mode network; LPFC: lateral prefrontal cortex; CEN: central executive network; MPFC: medial prefrontal cortex; p-FDR: *p*-value corrected for false discovery rate).

Network Seed	Target	T-Score	p-FDR
	**Depressed patients > HC**		
Left LP (DMN)	Left LPFC (CEN)	3.6	0.03
	MPFC (DMN)	3.1	0.03
	**Depressed FTD > HC**		
Left LP (DMN)	Left LPFC (CEN)	4.1	0.01
	MPFC (DMN)	2.7	0.05
	**Depressed PD > HC**		
Left LP (DMN)	Left LPFC (CEN)	2.3	0.05
	MPFC (DMN)	2.8	0.05

## Data Availability

The datasets generated and/or analyzed during the current study are available from the corresponding author on reasonable request.
